# Gut microbiota and metabolites in patients with COVID-19 are altered by the type of SARS-CoV-2 variant

**DOI:** 10.3389/fmicb.2024.1358530

**Published:** 2024-03-05

**Authors:** Yoshihiro Yokoyama, Tomoko Ichiki, Tsukasa Yamakawa, Yoshihisa Tsuji, Koji Kuronuma, Satoshi Takahashi, Eichi Narimatsu, Akio Katanuma, Hiroshi Nakase

**Affiliations:** ^1^Department of Gastroenterology and Hepatology, Sapporo Medical University School of Medicine, Sapporo, Japan; ^2^Department of General Medicine, Shiga University of Medical Science, Otsu, Shiga, Japan; ^3^Department of General Medicine, Sapporo Medical University School of Medicine, Sapporo, Japan; ^4^Department of Respiratory Medicine and Allergology, Sapporo Medical University School of Medicine, Sapporo, Japan; ^5^Department of Infection Control and Laboratory Medicine, Sapporo Medical University School of Medicine, Sapporo, Japan; ^6^Department of Intensive Care Medicine, Sapporo Medical University School of Medicine, Sapporo, Japan; ^7^Center for Gastroenterology, Teine-Keijinkai Hospital, Sapporo, Japan

**Keywords:** fecal calprotectin, metabolome, microbiome, short-chain fatty acids, Omicron

## Abstract

**Introduction:**

Patients with COVID-19 have dysbiosis of the intestinal microbiota with altered metabolites in the stool. However, it remains unclear whether the differences among SARS-CoV-2 variants lead to differences in intestinal microbiota and metabolites. Thus, we compared the microbiome and metabolome changes for each SARS-CoV-2 variant in patients with COVID-19.

**Materials and methods:**

We conducted a multicenter observational study of patients with COVID-19 and performed fecal microbiome, metabolome, and calprotectin analyses and compared the results among the different SARS-CoV-2 variants.

**Results:**

Twenty-one patients with COVID-19 were enrolled and stratified according to the SARS-CoV-2 strain: six with the Alpha, 10 with the Delta, and five with the Omicron variant. Fecal microbiome analysis showed that α-diversity was reduced in the order of the Omicron, Delta, and Alpha variants (*p* = 0.07). Linear discriminant analysis revealed differences in the abundance of short-chain fatty acid-producing gut microbiota for each SARS-CoV-2 variant. Fecal metabolome analysis showed that the Omicron and Delta variants had markedly reduced propionic and lactic acid levels compared to the Alpha strain (*p* < 0.05).

**Conclusion:**

The intestinal microbiota of patients with COVID-19 varies depending on the SARS-CoV-2 variant. Dysbiosis of the intestinal microbiota due to differences in SARS-CoV-2 variants causes a decrease in intestinal short-chain fatty acids.

## 1 Introduction

Coronavirus disease 2019 (COVID-19) is an infectious disease that was first reported in December 2019 and has since become a global pandemic. Patients with COVID-19 are known to have a high frequency of gastrointestinal symptoms such as abdominal pain, diarrhea, nausea and vomiting, and anorexia (Gu et al., 2020). COVID-19 patients with gastrointestinal symptoms have a more severe phenotype and are associated with poor prognosis (Hayashi et al., 2021; Bishehsari et al., 2022). One mechanism by which severe acute respiratory syndrome coronavirus 2 (SARS-CoV-2) infects human cells is via the angiotensin-converting enzyme 2 (ACE2)-mediated pathway (Penninger et al., 2021). ACE2 is highly expressed in intestinal epithelial cells, such as those in the small intestine and colon (Penninger et al., 2021), and previous studies have shown that SARS-CoV-2 is able to infect human cells of the gastrointestinal tract (Carvalho et al., 2020; Cheung et al., 2021; Jiao et al., 2021). ACE2 regulates human gut microbiota by regulating antimicrobial peptides in the small intestine (Hashimoto et al., 2012). Patients with COVID-19 are known to have altered gut microbiota depending on the severity of the illness, with dysbiosis being more significant in more severe cases (Chakraborty et al., 2022). Our institution has reported markedly decreased gene expression of ACE2 in the small intestine of patients with severe COVID-19, followed by impaired tryptophan metabolism (Yokoyama et al., 2022). Furthermore, studies analyzing the intestinal metabolites in patients with COVID-19 have shown a decrease in short-chain fatty acids such as butyric acid (Zhang et al., 2022). However, the differences between each SARS-CoV-2 variant in the gut microbiome and the metabolome of patients with COVID-19 remain unclear.

In this study, we compared the microbiome and metabolome changes for each SARS-CoV-2 variant in patients with COVID-19. We found that the intestinal microbiota was altered by SARS-CoV-2 variants, defined as Alpha, Delta, and Omicron strains and that the number of short-chain fatty acid-producing bacteria was particularly reduced. Furthermore, metabolomic analysis revealed that a decrease in short-chain fatty acids occurred with a decrease in the intestinal microbiota.

## 2 Materials and methods

### 2.1 Patients and sample collection

We included adult patients admitted at Sapporo Medical University and at Teine-Keijinkai hospital who showed positive results for SARS-CoV-2 infection by real-time PCR or antigen testing of nasopharynx or sputum samples. A positive real-time PCR result was defined as a cycle threshold value of 40 or lower, and a positive antigen test result was defined as a quantitative value of 100 pg./mL or higher. We divided the study subjects into critical and non-critical groups according to a previous report (Yokoyama et al., 2022). Patient backgrounds and hematological findings were obtained from their medical records.

Serum samples were leftover blood from routine blood tests and stored at −80°C. Stool samples were collected from patients and stored at 4°C for microbiome analysis and − 80°C for metabolomic analysis.

### 2.2 Fecal microbiome analysis

Genomic DNA from fecal samples was extracted using NucleoSpin^®^ DNA Stool (Macherey-Nagel, Duren, Germany) according to the manufacturer’s instructions and quantified using a Qubit 4 Fluorometer (Promega, Madison, WI, United States). 16S rRNA gene sequencing PCR and data processing were performed as previously described (Ariyoshi et al., 2021). Taxonomic classification for the microbiome was calculated by comparing all amplicon sequence variants with SILVA 138.1 using the q2-feature-classifier (Bokulich et al., 2018). α-diversity and β-diversity were calculated using QIIME2 (Hall and Beiko, 2018). Principal coordinate analysis (PCoA) was applied to distance matrices to create a two-dimensional plot. We used the linear discriminant analysis (LDA) effect size (LEfSe) approach to identify bacterial taxa that differed significantly between the critical and non-critical groups (Segata et al., 2011). Taxa groups with >3 log 10 LDA scores were considered significantly enriched at a *p*-value of <0.05.

### 2.3 Fecal metabolome analysis

Approximately 30–50 mg of feces were mixed with 500 μL of Milli-Q water containing internal standards (H3304-1002, HMT, Tsuruoka, Yamagata, Japan). The mixture was centrifuged at 2,300 × g and 4°C for 5 minutes, after which 80 μL of the supernatant was centrifugally filtered through a Millipore 5-kDa cutoff filter (ULTRAFREE MC PLHCC, HMT) at 9,100 × *g* and 4°C for 120 min to remove macromolecules. Subsequently, the filtrate was evaporated to dryness under vacuum and reconstituted in 20 μL of Milli-Q water for metabolome analysis. Metabolome analysis was conducted according to the HMT Basic Scan package using capillary electrophoresis time-of-flight mass spectrometry (CE-TOF-MS) based on previously described methods (Ohashi et al., 2008; Ooga et al., 2011). Briefly, CE-TOF-MS analysis was performed using an Agilent CE capillary electrophoresis system equipped with an Agilent 6,210 TOF mass spectrometer (Agilent Technologies, Inc., Santa Clara, CA, United States). The systems were controlled by Agilent G2201AA ChemStation software version B.03.01 (Agilent Technologies) and connected by a fused silica capillary (50 μm i.d. × 80 cm total length) with commercial electrophoresis buffer (H3301-1001 and I3302-1023 for cation and anion analyses, respectively, HMT) as the electrolyte. The spectrometer was scanned from m/z 50 to 1,000, and peaks were extracted using MasterHands automatic integration software (Keio University, Tsuroka, Yamagata, Japan) to obtain peak information, including m/z, peak area, and migration time (Sugimoto et al., 2010). Signal peaks corresponding to isotopomers, adduct ions, and other product ions of known metabolites were excluded, and the remaining peaks were annotated according to the HMT metabolite database based on their m/z values and migration times. The areas of the annotated peaks were normalized to the internal standards and sample amounts to obtain the relative levels of each metabolite. Hierarchical cluster analysis and principal component analysis (Yamamoto et al., 2014) were performed by HMT proprietary MATLAB and R programs, respectively.

### 2.4 Fecal calprotectin analysis

Fecal calprotectin (FCP) levels were calculated using a sandwich enzyme immunoassay kit (Calprotectin ELISA Kit, Calprotectin Mochida^®^). In healthy control, the 95% confidence interval for FCP ranges from 0 to 94 μg/g (mean value = 43.0 μg/g, SD = ±26.0 μg/g) (Nancey et al., 2013). We defined a result as 30 μg/g if the measured FCP value was less than 30 μg/g, which is below the sensitivity limit of the assay.

### 2.5 Statistics

We analyzed normally distributed continuous variables using an unpaired Student’s *t*-test, and non-normally distributed data using the Wilcoxon rank-sum test. For comparison of values among COVID-19 variants, one-way ANOVA with Tukey’s test was applied. A Kruskal-Wallis test was applied for α-diversity and LEfSe and *p*-values for β-Diversity were calculated by using permutational analysis of variance.

We defined two-sided *p*-values less than 0.05 as indicate statistical significance. All analyses were performed using JMP, version 15 (SAS Institute, Cary, NC, United States).

### 2.6 Study approval

This multicenter, prospective, observational study was undertaken at Sapporo Medical University, Sapporo, Japan, and was approved by the ethical committee of Sapporo Medical University (IRB number: 332-111). This study is part of a comprehensive clinical trial registered with the University Hospital Medical Information Network Clinical Trial Registry (UMIN-CTR: 000046106). We obtained written informed consent for all the patients participating in the study.

To ensure the accuracy and completeness of the data and the fidelity of the protocol, all authors contributed to the collection and analysis of the data. This study was conducted in accordance with the principles of the Declaration of Helsinki.

## 3 Results

### 3.1 Study patients

We obtained informed consent from 53 patients with COVID-19 between July 2021 and January 2023. Of these, 21 were positive for COVID-19 variants: six for Alpha, 10 for Delta, and five for Omicron variants. The median age was 49.0 years (Alpha/Delta/Omicron: 44.5/50.0/45.0), body mass index was 26.7 (28.8/25.6/27.9) kg/m^2^, and disease severity was classified as mild/moderate/severe/critical and 12/2/2/5 patients. The period between the SARS-CoV-2 test positive and collection of fecal samples was 4 days (2–12), for each variant: Alpha 5 (3–9), Delta 4.5 (2–12) and Omicron 4 (3–10), respectively. Gastrointestinal symptoms included diarrhea in four patients (19.0%), anorexia in four patients (19.0%), abdominal pain in three patients (14.3%), and nausea in three patients (14.3%). Patient characteristics are shown in Table 1.

**Table 1:** Clinical characteristics of the patients with COVID-19 included in the study.

	All (*n* = 21)	Alpha (*n* = 6)	Delta (*n* = 10)	Omicron (*n* = 5)
General characteristics				
Age, y	49.0 ± 15.4	44.5 ± 15.6	50.0 ± 14.8	45.0 ± 18.5
Gender, Male	14 (66.7)	5 (83.3)	6 (60.0)	3 (60.0)
Body mass index, kg/m^2^	26.7 ± 5.4	28.8 ± 4.6	25.6 ± 5.9	27.9 ± 5.1
Current smoking	5 (23.8)	2 (33.3)	2 (20.0)	1 (20.0)
Covid-19 disease severity category				
Mild	12 (57.1)	3 (50.0)	6 (60.0)	3 (60.0)
Moderate	2 (9.5)	1 (16.7)	1 (10.0)	0 (0)
Severe	3 (14.3)	0 (0)	2 (20.0)	1 (20.0)
Critical	4 (19.0)	2 (33.3)	1 (10.0)	1 (20.0)
**Vaccination**	6 (28.6)	1 (16.7)	3 (30.0)	2 (40.0)
**Period between SARS-CoV-2 test positive and sample collection, days**	4 (2–12)	5 (3–9)	4.5 (2–12)	4 (3–10)
Comorbidity				
Hypertension	8 (38.1)	3 (50.0)	4 (40.0)	1 (20.0)
Diabetes mellitus	4 (19.0)	1 (16.7)	2 (20.0)	1 (20.0)
Hyperlipidemia	3 (14.3)	3 (50.0)	0 (0)	0 (0)
COPD	2 (9.5)	1 (16.7)	0 (0)	1 (20.0)
Symptoms at hospitalization				
High fever >37.5°C	10 (47.6)	2 (33.3)	6 (60.0)	2 (40.0)
General fatigue	10 (47.6)	3 (50.0)	6 (60.0)	1 (20.0)
Cough	13 (61.9)	4 (66.7)	7 (70.0)	2 (40.0)
Diarrhea	4 (19.0)	2 (33.3)	2 (20.0)	1 (20.0)
Appetite loss	4 (19.0)	0 (0)	3 (30.0)	1 (20.0)
Abdominal pain	3 (14.3)	1 (7.7)	1 (10.0)	1 (20.0)
Nausea	3 (14.3)	2 (15.4)	1 (10.0)	0 (0)
Therapeutics after admission				
Steroids	14 (66.7)	5 (83.3)	7 (70.0)	2 (40.0)
Antiviral antibodies	12 (57.1)	3 (50.0)	6 (60.0)	3 (60.0)
Anti-inflammatory drugs	14 (66.7)	5 (83.3)	8 (80.0)	1 (20.0)
Anti-SARS-CoV-2 antibody cocktail	7 (33.3)	3 (23.1)	3 (30.0)	1 (20.0)

Data are expressed as the mean ± SD or number (percentage). Percentages may not add up to 100% due to rounding or overlap. COPD, chronic obstructive pulmonary disease.

### 3.2 Fecal microbiome analysis

**Figure 1:**
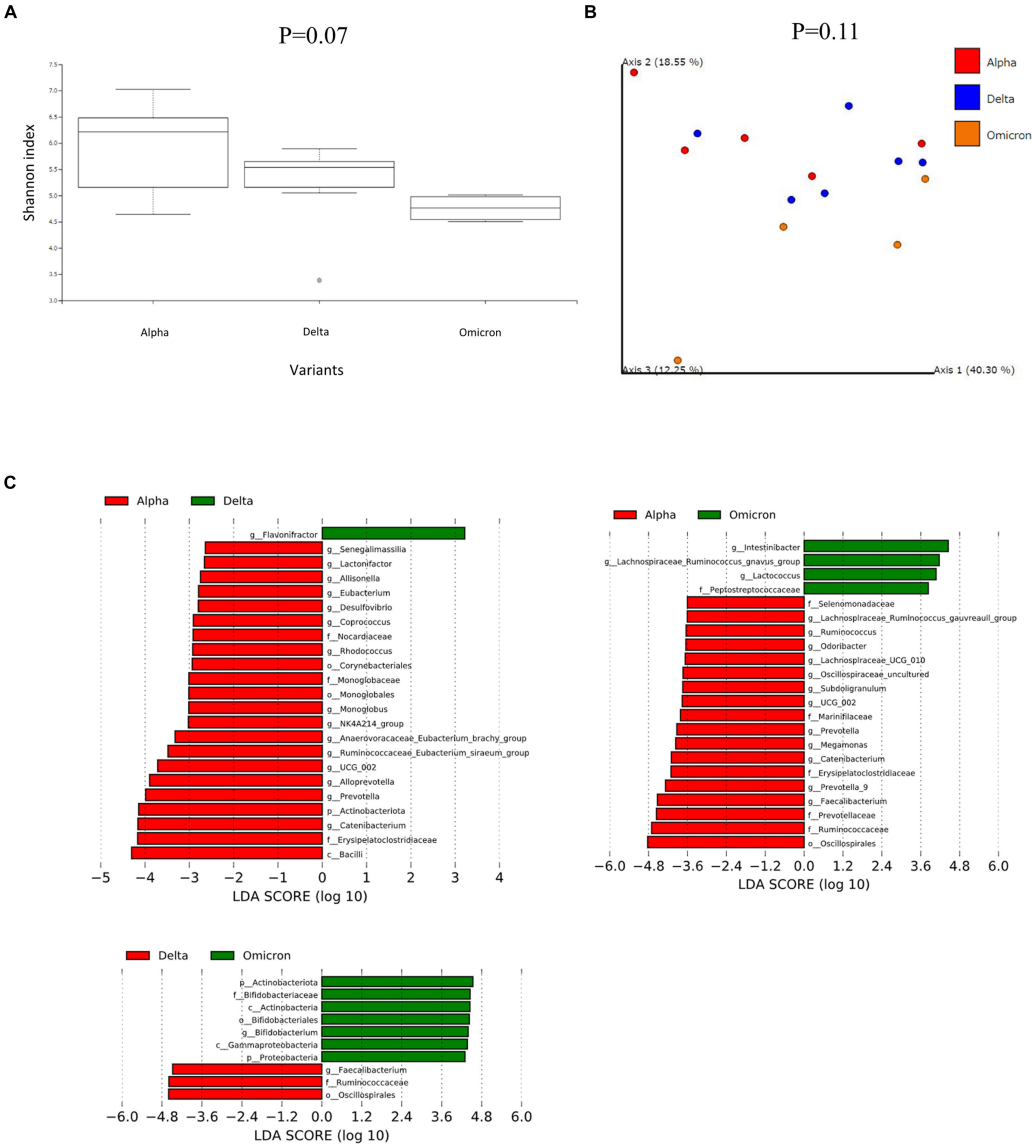
Fecal microbiome analysis comparing each infected variants in patients with COVID-19. **(A)** The α-diversity represented by the Shannon index. The vertical axis represents Shannon’s index. Box-and-whisker plots show the median, 25th percentile, and 75th percentile, with whiskers extending to the minimum and maximum values. Data were analyzed using the Kruskal-Wallis test. Statistical significance was set at *p* < 0.05. **(B)** β-diversity evaluated by the unweighted UniFrac distances applied for principal coordinates analysis (PCoA). Statistical analysis was performed using permutational analysis of variance (PERMANOVA). Statistical significance was set at *p* < 0.05. **(C)** Enriched gut microbiota constituents were identified using the linear discriminant analysis (LDA) effect size (LEfSe). The histogram of the LDA scores with (log 10) values >3 and *p* < 0.05 revealed the most differentially abundant taxa among the different reproductive stages.

We analyzed the fecal microbiome of patients with COVID-19 and classified them according to SARS-CoV-2 variants (Figure 1). α-diversity, as represented by the Shannon index, decreased in the order of the microbiomes of patients infected with Alpha, Delta, and Omicron variants (*p* = 0.07, Figure 1A). β-diversity evaluated by the unweighted UniFrac distances applied for PCoA was analyzed for each SARS-CoV-2 variant, no significant differences were obtained despite a slight divergence between Omicron and other strains (*p* = 0.11, Figure 1B). Next, we used LEfSe analysis to identify bacterial taxa that were significantly different in abundance between each SARS-CoV-2 variant (Figure 1C). First, a comparison of the Alpha and Delta strains showed that short-chain fatty acid-producing bacteria, such as *Catenibacterium*, *Ruminococcus*, and *Eubacterium,* were more abundant in the microbiome of patients infected with the Alpha strain than with the Delta strain. *Oscillospirales*, *Faecalibacterium*, *Catenibacterium*, and *Subdoligranulum*, which also produce short-chain fatty acids, mainly butyric acid, were more abundant in the microbiome of patients infected with the Alpha strain than Omicron. Finally, comparing the Delta and Omicron strains, *Oscillospirales*, *Ruminococcus*, and *Faecalibacterium* were abundant in the Delta strain, while *Bifidobacterium* was abundant in the Omicron strain, indicating that both strains produce short-chain fatty acids but differ in the composition. These data suggest that the gut microbiota in patients with COVID-19 varied depending on the SARS-CoV-2 variants and that the changes were mainly due to the different compositions of the bacteria producing short-chain fatty acids.

**Figure 2:**
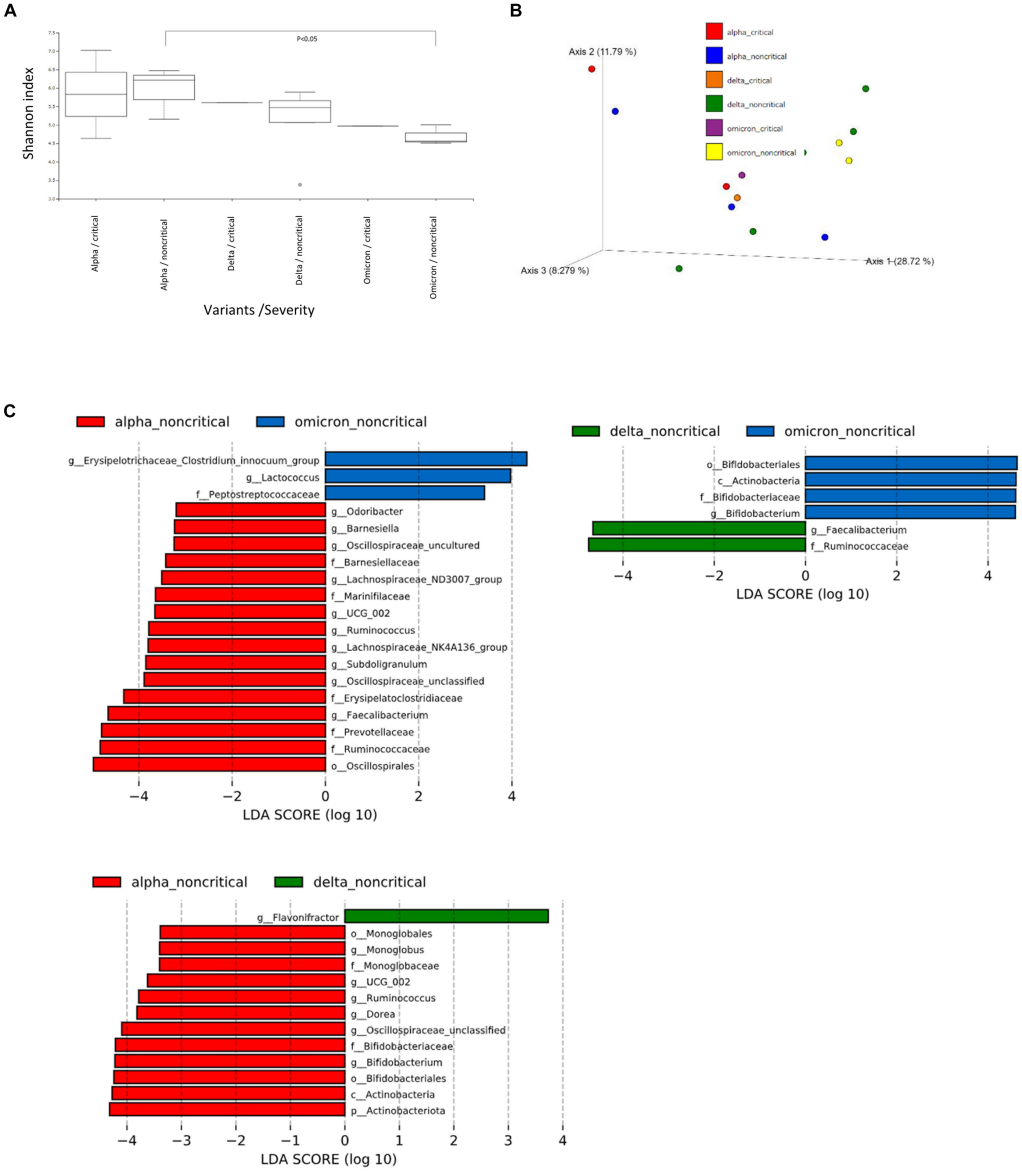
Fecal microbiome analysis of COVID-19 patients compared by infected variants and critical/noncritical group. **(A)** The α-diversity represented by the Shannon index classified by SARS-CoV-2 variant and severity. The vertical axis represents Shannon’s index. Box-and-whisker plots show the median, 25th percentile, and 75th percentile, with whiskers extending to the minimum and maximum values. Data were analyzed using the Kruskal-Wallis test. Statistical significance was set at *p* < 0.05. **(B)** β-diversity evaluated by the unweighted UniFrac distances applied for principal coordinates analysis (PCoA) classified by SARS-CoV-2 variant and severity. **(C)** The enriched gut microbiota constituents were identified by linear discriminant analysis (LDA) effect size (LEfSe) and classified by the SARS-CoV-2 variant and severity. The histogram of the LDA scores with (log 10) values >3 and *p* < 0.05 revealed the most differentially abundant taxa among the different reproductive stages.

We then performed further analysis of the microbiome data for each SARS-CoV-2 variant, classifying them into critical and non-critical cases (Figure 2). Interestingly, in the non-critical group, the microbiome of patients with COVID-19 infected with omicron strains showed a significant reduction in α-diversity compared to Alpha strains (Figure 2A). However, no characteristic differences in β-diversity were found when classified as critical or non-critical (Figure 2B). LEfSe analysis of the non-critical group showed a pattern similar to that in Figure 1C (Figure 2C). These results indicate that patients with COVID-19 infected with the Omicron strain, at least in non-critical cases, have a clearly altered microbiota compared to patients infected with the Alpha strain.

### 3.3 Fecal metabolome analysis

**Figure 3:**
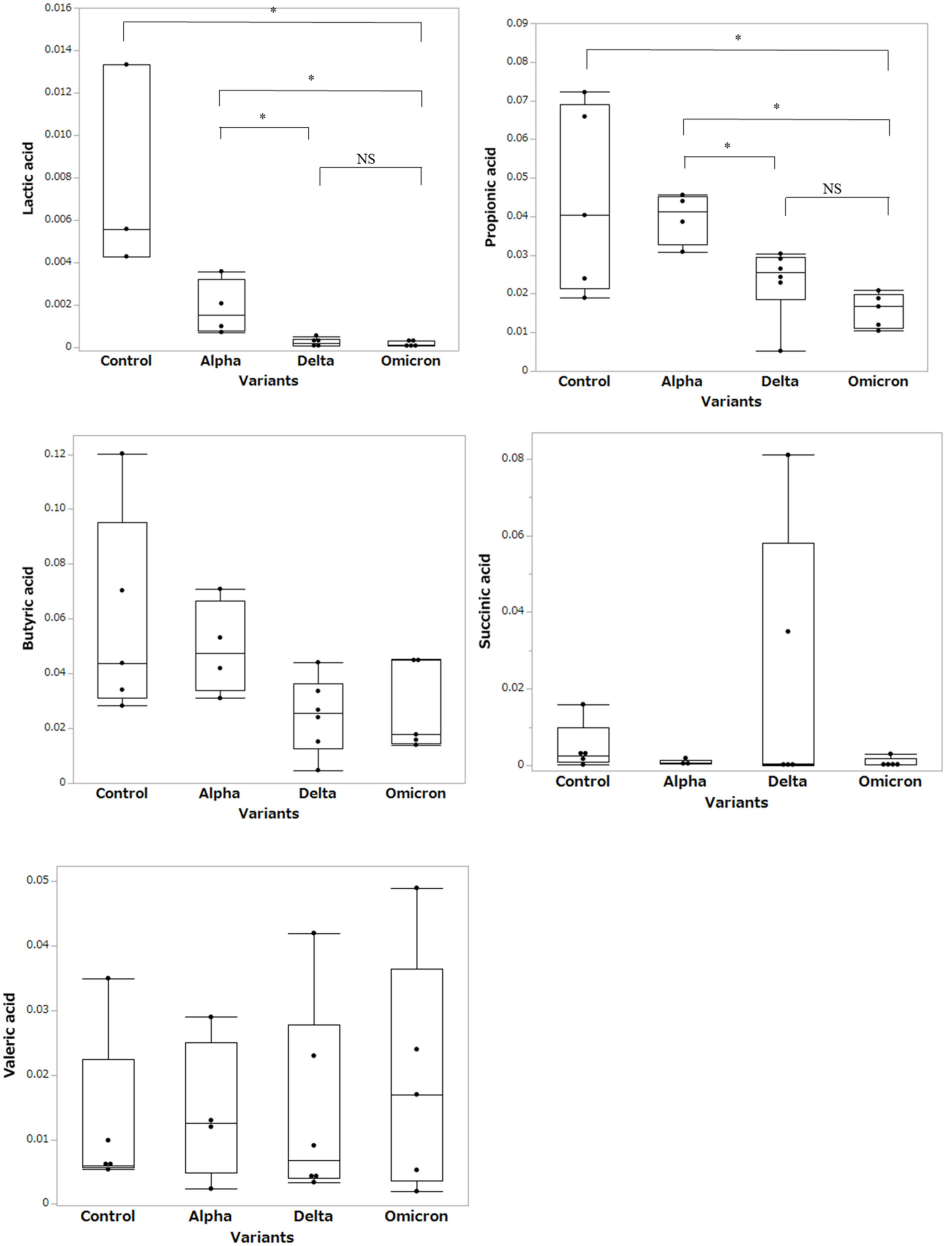
Comparison of short-chain fatty acids in stool metabolites classified by the SARS-CoV-2 variants. Statistical analysis was performed using a one-way ANOVA with Tukey’s test. Statistical significance is indicated by asterisks at *P* < 0.05.

**Figure 4:**
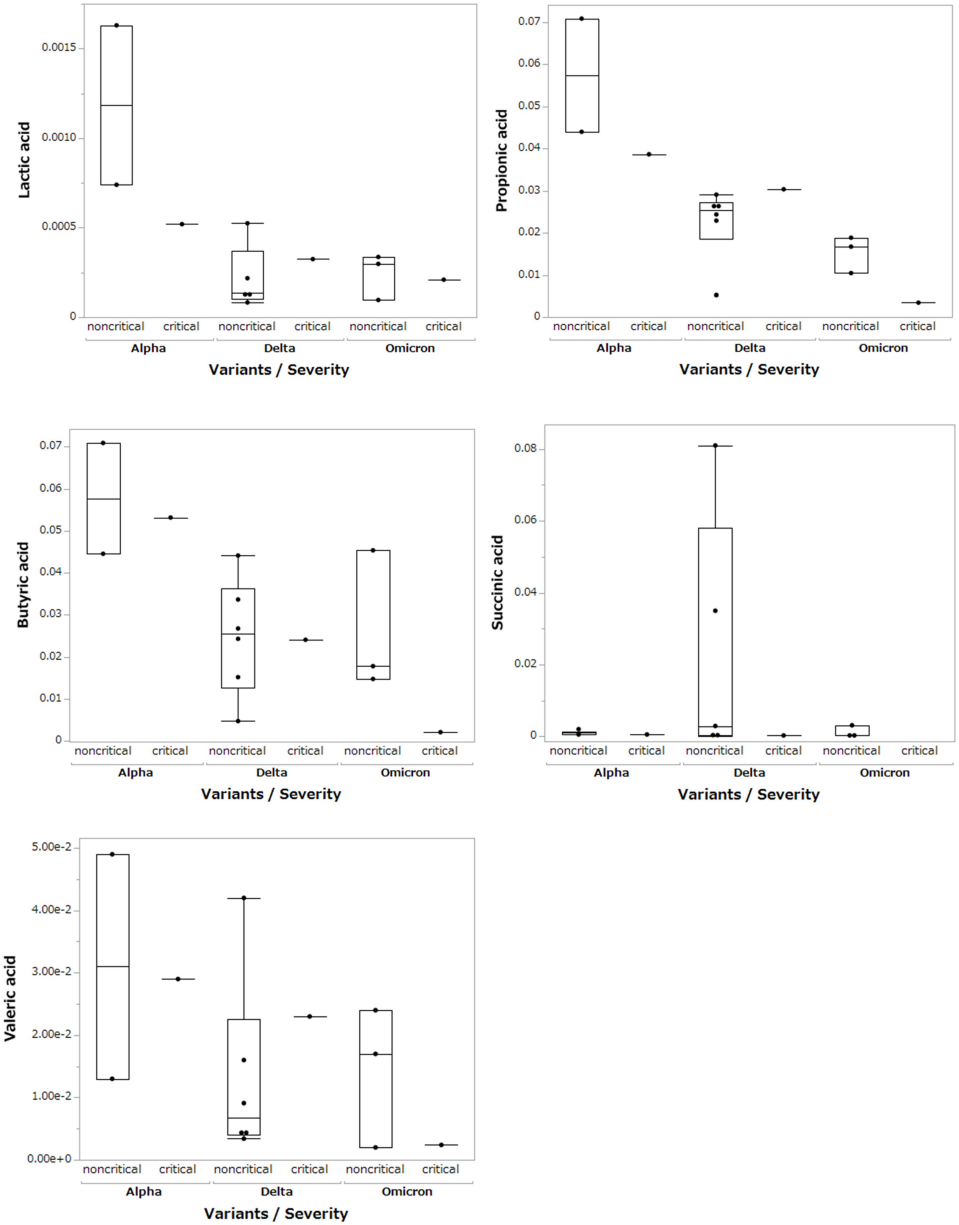
Comparison of short-chain fatty acids in stool metabolites classified by SARS-CoV-2 variant and severity.

Based on the results of the fecal microbiome analysis, we focused on short-chain fatty acids in the stool metabolome analysis. For comparison with COVID-19 patients, we used data from a group of healthy controls not affected by COVID-19 (*n* = 5). Compared to data from healthy controls, lactic acid and propionic acid were lower in COVID-19 patients, with only the Omicron strain showing statistically significant differences (*p* < 0.05; Figure 3). In a comparison among the SARS-CoV-2 strains, lactic acid and propionic acid were markedly decreased in the stool metabolites of patients infected with the Delta and Omicron strains compared to the Alpha strain (*p* < 0.05). Butyric acid was decreased in the stool of patients infected with the Delta and Omicron strains compared with the Alpha strain; however, the difference was not significant. Succinic and valeric acid levels did not vary among the strains. We further analyzed the metabolomic data for each SARS-CoV-2 variant and classified them into critical and non-critical cases (Figure 4). Lactic acid and propionic acid levels were decreased in the stool of patients with COVID-19 infected with the Delta and Omicron strains compared to those infected with the Alpha strain. These data provide direct evidence that SARS-CoV-2 variants contribute to the production of short-chain fatty acids in stool by influencing the gut microbiome.

### 3.4 Fecal calprotectin analysis

**Figure 5:**
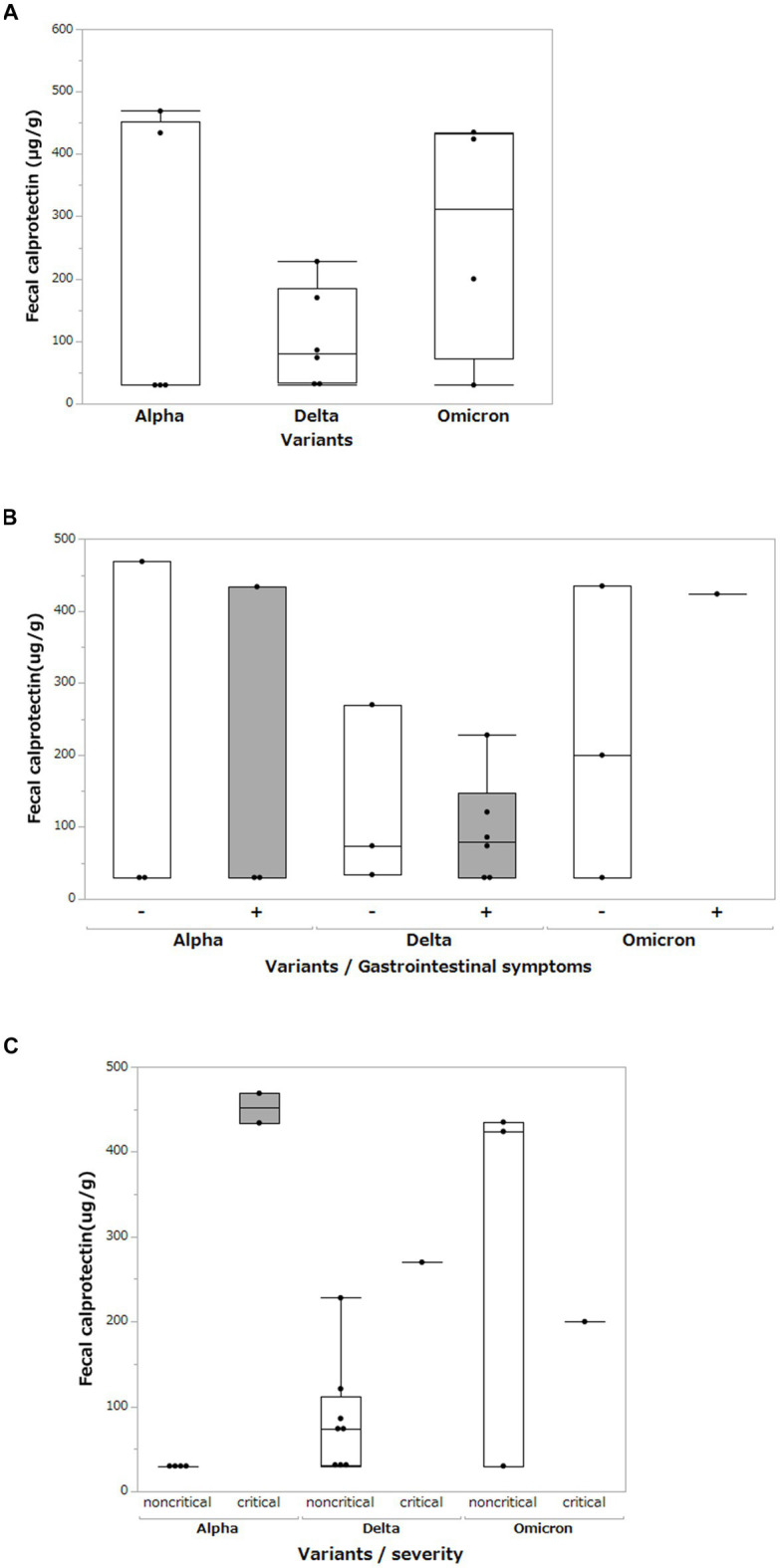
Fecal calprotectin levels between different SARS-CoV-2 variants. **(A)** Comparison of fecal calprotectin levels between the Alpha, Delta, and Omicron SARS-CoV-2 variants. Box-and-whisker plots show the median, 25th percentile, and 75th percentile, with whiskers extending to the minimum and maximum values. Each dot represents an individual value. Statistical analyses were performed using the Wilcoxon rank-sum test. Statistical significance was set at P < 0.05. If the measured FCP value was under 30 μg/g, which is less than the sensitivity limit of the assay, the result was defined as 30 μg/g. **(B)** Comparison of fecal calprotectin levels among the Alpha, Delta, and Omicron SARS-CoV-2 variants with and without gastrointestinal symptoms (diarrhea and abdominal pain). **(C)** Comparison of fecal calprotectin levels among the Alpha, Delta, and Omicron variants of SARS-CoV-2 and the severity of COVID-19.

We measured the FCP levels in the stool of patients with COVID-19 and compared them for each SARS-CoV-2 variant (Figure 5A). Mean FCP levels in patients infected with the Alpha, Delta, and Omicron variants were 198.6 μg/g (SD ±231.2 μg/g), 103.7 μg/g (SD ±79.2 μg/g), and 272.3 μg/g (SD ±194.4 μg/g), respectively. There were no significant differences in FCP levels among these variants. We analyzed FCP levels in patients with and without gastrointestinal symptoms such as diarrhea or abdominal pain (Figure 5B). The FCP level was not affected by the presence or absence of abdominal symptoms. We further analyzed FCP levels according to the SARS-CoV-2 variant and severity of COVID-19, classifying them as critical or non-critical (Figure 5C). The data showed that the FCP levels were higher in critical cases than in non-critical cases, regardless of the SARS-CoV-2 variant. Interestingly, while FCP levels were not elevated in the non-critical group of the Alpha strain, markedly elevated FCP levels were observed in the non-critical group of the Delta and Omicron strains.

## 4 Discussion

This study showed that the gut microbiota of patients with COVID-19 differed according to the SARS-CoV-2 variant. α-diversity was decreased in the order of Omicron, Delta, and Alpha strains, with a significant difference between the Omicron and Alpha strains, especially in the non-critical group. We also confirmed a decrease in short-chain fatty acids in the stool owing to a decrease in short-chain fatty acid-producing bacteria. In addition, FCP was found to be more elevated in the fecal samples of patients with COVID-19 infected with the Omicron strain than in those infected with the Alpha and Delta strains in a comparison of the non-critical groups.

To our knowledge, this is the first comparative analysis of gut microbiota and metabolites among patients infected with three different SARS-CoV-2 variants. COVID-19 causes gastrointestinal symptoms during the early stages of the disease (Wiersinga et al., 2020). The mechanism of gastrointestinal symptoms in COVID-19 is speculated to involve the abundant expression of ACE2, the entry portal of SARS-CoV-2 into the gastrointestinal tract (Cheung et al., 2021; Jiao et al., 2021). Studies on intestinal bacteria in patients with COVID-19 showed that intestinal bacteria, including *Faecalibacterium prausnitzii*, *Eubacterium rectale*, and several species of bifidobacteria, decreased with disease severity (Yeoh et al., 2021). Metabolomic analysis of patients with COVID-19 showed decreased short-chain fatty acid and L-isoleucine biosynthesis in the gut microbiota, which was correlated with disease severity (Zhang et al., 2022). Furthermore, our laboratory reported a marked decrease in indole 3-propionate, a tryptophan metabolite, in patients with severe COVID-19 (Yokoyama et al., 2022). However, the effects of SARS-CoV-2 variants on intestinal microbiota and intestinal metabolites have not been elucidated (Zhang et al., 2023).

First, we analyzed the gut microbiota by classifying SARS-CoV-2 variants into Alpha, Delta, and Omicron strains and found that α-diversity decreased in patients infected with the Omicron strain. LEfSe analysis, which reflects the abundance of microbiota, showed that *Catenibacterium*, *Ruminococcus*, and *Eubacterium* were more abundant in the Alpha strain than in the Delta strain, and *Oscillospirales*, *Faecalibacterium*, *Catenibacterium*, and *Subdoligranulum* were more abundant in the Alpha strains than in Omicron strains. All these bacteria are known to produce short-chain fatty acids such as propionic acid and butyric acid (Ohira et al., 2017; Fernández-Veledo and Vendrell, 2019; Van Hul et al., 2020; Yang et al., 2021; Hazan et al., 2022; Xie et al., 2022; Huang et al., 2023). One hypothesis as to why the composition of the gut microbiota was different for each SARS-CoV-2 variant may explain the response of SARS-CoV-2 to the gut epithelium and ACE2. Using intestinal organoids, Jang et al. reported that the infectivity of SARS-CoV-2 for the intestinal epithelium was strongest in the Omicron strain and that the infectivity correlated with ACE2 expression levels (Jang et al., 2022). Furthermore, *in vivo* experiments showed that the Omicron variant is susceptible to binding to the human ACE2 receptor (Lupala et al., 2022). Based on these basic data, we considered that mutant strains of SARS-CoV-2 (Omicron and Delta) bind more strongly to the ACE2 receptor than Alpha strains, thereby reducing ACE2 expression in the intestinal tract and causing dysbiosis (Hashimoto et al., 2012). Experimental studies using animal and *in vivo* models are required to confirm this mechanism. Since short-chain fatty acids play a role in regulating the immune response in the intestinal tract (Akhtar et al., 2022), a decrease in short-chain fatty acids could induce intestinal inflammation.

The FCP level correlates with disease severity in patients with COVID-19 (Effenberger et al., 2020; Ojetti et al., 2020), and our previous data showed a marked increase in FCP in critical cases (Yokoyama et al., 2022). The present study also examined non-critical cases and found that FCP levels were higher in patients infected with the Omicron strain than in those infected with the Alpha strain. Furthermore, the α-diversity of the microbiome was lower in patients infected with the Omicron strain than in patients infected with the Alpha strain. These facts suggest that the Omicron strains may cause dysbiosis and inflammation of the gastrointestinal tract, regardless of disease severity. Further studies are needed to determine whether differences in SARS-CoV-2 variants, particularly in the reduction of short-chain fatty acids in the Delta and Omicron strains, induce intestinal inflammation and their clinical significance.

This study has several limitations. First, this study is the lack of the WHO classification of patients by disease severity. As previously reported, the microbiome and metabolome of patients with COVID-19 correlate with disease severity (Yeoh et al., 2021; Yokoyama et al., 2022). In this study, although we could classify non-critical cases by SARS-CoV-2 variant, our sample size was small, and we could not fully examine critical cases. Thus, statistical tests could not be performed and the interpretation of the results was difficult. Second, because this study was a multicenter study in Japan, only Japanese patients were enrolled in the study, so comparisons between races were not performed. Third, vaccination affects the severity and the intestinal microbiota in patients with COVID-19, but we could not examine in detail the timing of vaccination or the type of vaccine in this study. Finally, we could not compare the healthy control and COVID-19 groups in our microbiome analysis because there were no adequate fecal samples from healthy individuals during the COVID-19 pandemic.

In conclusion, we found that the type of SARS-CoV-2 variant causes changes in intestinal inflammation and bacterial diversity, as well as a decrease in fatty acids in the intestinal tract. SARS-CoV-2 continues to mutate; however, the mechanism by which it affects the gastrointestinal tract has not been elucidated. Further studies on the disruption of the intestinal immune system by SARS-CoV-2 and changes in the intestinal microbiota will play an important role in treating new viral infections that may occur.

## Data availability statement

The datasets presented in this study can be found in online repositories. The names of the repository/repositories and accession number(s) can be found at: https://www.ncbi.nlm.nih.gov/bioproject/PRJDB13936/.

## Ethics statement

The studies involving humans were approved by the Ethical Committee of Sapporo Medical University. The studies were conducted in accordance with the local legislation and institutional requirements. The participants provided their written informed consent to participate in this study.

## Author contributions

YY: Conceptualization, Data curation, Methodology, Writing – original draft, Writing – review & editing. TI: Data curation, Formal analysis, Writing – review & editing. TY: Data curation, Writing – review & editing. YT: Supervision, Writing – review & editing. KK: Supervision, Writing – review & editing. ST: Supervision, Writing – review & editing. EN: Supervision, Writing – review & editing. AK: Supervision, Writing – review & editing. HN: Conceptualization, Funding acquisition, Supervision, Writing – review & editing.
